# Differential Effects of Phenanthrene and Its Chlorinated Congeners on Hormone Production and Mitochondrial Function in Ovarian Granulosa Cells

**DOI:** 10.3390/toxics14040313

**Published:** 2026-04-07

**Authors:** Genevieve A. Perono, Laiba Jamshed, Rohita Dutt, Reese S. Cameron, James J. Petrik, Philippe J. Thomas, Alison C. Holloway

**Affiliations:** 1 Department of Obstetrics and Gynecology, McMaster University, Hamilton, ON L8S 4L8, Canada; peronog@mcmaster.ca (G.A.P.);; 2 Department of Biomedical Sciences, University of Guelph, Guelph, ON N1G 2W1, Canada; jpetrik@uoguelph.ca; 3 Wildlife and Landscape Science Directorate, National Wildlife Research Center, Environment and Climate Change Canada, Ottawa, ON K1A 0H3, Canada; philippe.thomas@ec.gc.ca

**Keywords:** polycyclic aromatic hydrocarbons (PAH), ClPAH, phenanthrene, endocrine disruption, mitochondria, apoptosis, autophagy, ovary

## Abstract

Halogenated derivatives of polycyclic aromatic hydrocarbons (PAHs), such as chlorinated PAHs (ClPAHs), are an emerging class of contaminants that are being detected in the environment as well as in wildlife and human populations. Previous studies have shown that chemical substitution of PAHs, including chlorination, may alter the toxicity of parent PAHs; however, whether chlorination affects their endocrine-disrupting potential remains unexplored. In this study, we examined the effects of phenanthrene (Phe), one of the most prevalent PAHs, and its chlorinated congeners, 9-chlorophenanthrene (9ClPhe) and 9,10-dichlorophenanthrene (9,10Cl_2_Phe), on hormone production in granulosa cells, key hormone-secreting cells of the ovary. We observed that Phe and its chlorinated congeners differentially altered anti-Müllerian hormone (AMH), estradiol (E2), and progesterone (P4) secretion. Since mitochondria are central to steroidogenesis, we further evaluated mitochondrial function. While Phe increased ATP production, both 9ClPhe and 9,10Cl_2_Phe increased ROS, decreased mitochondrial membrane potential, and reduced the expression of markers for mitochondrial dynamics and mitophagy without altering ATP levels. We further tested impacts on cell fate and found that neither Phe nor its chlorinated congeners altered granulosa cell apoptosis. Together, these results suggest that chlorination of Phe leads to dose-dependent, differential effects on hormone production and mitochondrial pathways without inducing cell death in granulosa cells. This study highlights the potential adverse impacts of ClPAH exposure on ovarian follicle development and female fertility by disrupting steroidogenesis and mitochondrial quality control.

## 1. Introduction

Polycyclic aromatic hydrocarbons (PAHs) are compounds consisting of two or more aromatic rings made of carbon and hydrogen atoms, and are considered to be environmental contaminants [1]. While PAHs can be derived from natural sources, anthropogenic activities such as industrialization, urbanization, and electronic waste (e-waste) burning contribute to emissions of PAHs into the environment [2]. The toxic effects of PAHs are well-documented [3], with many exhibiting carcinogenic, immunotoxic, and adverse reproductive effects [2,3]. Most studies to date have focused on 16 PAHs identified as priority pollutants by the United States Environmental Protection Agency in 1976, although attention has shifted more recently towards the broader class of PAHs [4]. Phenanthrene (Phe), a 3-ring, low molecular weight PAH, is one of the most extensively studied PAHs given its widespread occurrence in the air, sediment, soil, and water near urban and industrialized areas [5,6,7]. Evidence from studies in aquatic vertebrates has shown that exposure to Phe causes reproductive deficits and endocrine disruption, including reductions in egg production, fertilization rates, and hatching success, along with increased larval malformations, impaired oocyte maturation, and altered hormone levels [5,8,9,10,11]. The endocrine-disrupting effects of PAHs, including Phe, have been reviewed elsewhere [12], and exposure to Phe has been linked to adverse reproductive outcomes like polycystic ovary syndrome (PCOS) or premature ovarian insufficiency (POI) [13]. Consistent with these findings, mice exposed to Phe *in utero* exhibited enlarged ovaries, increased antral and atretic follicle counts, and diminished circulating hormone levels [9]. Changes in hormone balance and endocrine disruption may be explained, in part, by perturbations in mitochondrial function. Mitochondria play a central role in steroidogenesis and follicle growth, key components of female fertility. Mitochondrial dysfunction has been linked to impaired fertility, PCOS, and POI [14,15,16]. Notably, mitochondria are also vulnerable targets of many environmental contaminants, including PAHs, and their halogenated derivatives [17,18,19].

Chlorinated phenanthrenes (ClPhe) have been detected in urban air pollution [20], around waste incineration and e-waste recycling sites [21,22], and in surface sediment near urban regions [23,24]. Occupational exposure studies, such as those conducted in coal-fired power plant workers, identified ClPhe as one of the most abundant halogenated PAHs detected in serum (1.60–30.4 ng/g lipid in females) [25]. However, for non-smokers and individuals without occupational exposure, dietary intake is considered the predominant route of exposure to PAHs [26,27]. Consistent with this, ClPhe and other ClPAHs have been detected in seafood [28,29], crops, and meats [27,30,31], as well as wildlife living near areas with high industrial activity [32], suggesting that exposure to ClPAHs may occur through ingestion of exposed food sources. A study measuring nine halogenated PAHs in vegetables, pork, and rice reported total concentrations ranging from 0.56 to 4.97 ng/g, with estimated daily dietary intake of 17.6–31.8 ng/kg body weight per day in females [31]. Compared to their parent PAHs, ClPAHs exhibit greater lipid partitioning and bioaccumulation potential, and given that some studies report that halogenation may alter the toxicological effects of parent PAHs [24,33,34,35], ClPAHs may pose significant risks to wildlife and human health [36]. Some chlorinated PAH derivatives have been shown to be stronger inducers of DNA damage and aryl hydrocarbon receptor (AhR) activity than their parent counterparts and these effects are influenced by the degree and position of the chlorine substituents [33]. Similarly, studies on chlorinated congeners of other PAHs like benzo[*a*]pyrene (BaP) or anthracene have demonstrated that the chlorinated congener led to greater oxidative stress and mitochondrial dysfunction in hepatic cells compared to their non-chlorinated counterparts [37,38].

Given that Phe has been shown to affect hormone production and impair mitochondrial function and fertility, the focus of this study was to examine and characterize the effects of Phe and its chlorinated congeners, 9-chlorophenanthrene (9ClPhe) and 9,10-dichlorophenanthrene (9,10Cl_2_Phe), on hormone production, mitochondrial function, and cell fate in granulosa cells, key cells that are critical to ovarian follicle development and hormone synthesis.

## 2. Materials and Methods

### 2.1. Cell Culture Maintenance and Treatment

Spontaneously immortalized granulosa cells (SIGCs), a rat granulosa cell line [39], were cultured (passages 13–18) in DMEM/Ham’s F-12 media with L-glutamine (Corning, Corning, NY, USA) supplemented with 10% (*v*/*v*) fetal bovine serum (FBS; Cytiva, Marlborough, MA, USA) and 2% (*v*/*v*) penicillin-streptomycin (Gibco, Waltham, MA, USA) on 100 mm culture dishes (Corning) in a humidified atmosphere of 95% air and 5% CO_2_ at 37 °C.

Cells were seeded at 250,000 cells/well in 6-well tissue culture plates (Falcon^®^, Corning, NY, USA) and exposed to 0 (vehicle, 0.1% DMSO), 0.01, 0.1, or 1 μM of phenanthrene (Phe), 9-chlorophenanthrene (9ClPhe), or 9,10-dichlorophenanthrene (9,10Cl_2_Phe) (Toronto Research Chemicals, North York, ON, Canada) (N = 6 independent experiments) in supplemented media for 24 h. These chlorinated congeners are among the most commonly detected halogenated PAHs in food and wildlife, and the concentrations selected for this study were based on environmentally and biologically relevant levels of Phe and chlorinated Phe reported in the serum of coal-fired power plant workers [25], seafood [24,29,40], crops and meat [27,28,30], and liver tissues of biota near oil sands regions in Alberta [32]. Following 24 h treatment of SIGCs, culture media was collected for hormone assessments and cells were collected to assess steady-state mRNA expression.

### 2.2. Steroid Hormone Assessment

Hormone levels following exposures to Phe and chlorinated Phe were quantified using commercially available kits, according to the manufacturer’s instructions. We measured anti-Müllerian hormone (AMH) (abx155189, Abbexa Ltd., Cambridge, UK), progesterone (P4) (KA0299, Abnova, Taipei City, Taiwan) and estradiol (E2) levels (ADI-900-008, Enzo Life Sciences, Farmingdale, NY, USA) in the spent culture media. The reported intra-assay and inter-assay coefficients of variation for all the ELISA kits ranged from 4.9–10% to 2.7–12%, respectively, and all measured samples were above the reported limits of detection and within the range of the standard curve for each assay. Absorbance values were obtained using the Synergy^TM^ H1 microplate reader (Agilent Technologies, Inc., Santa Clara, CA, USA) and were fit to a four-parameter logistic regression curve using the GainData^®^ ELISA calculator (arigo Biolaboratories Corp., Zhubei City, Taiwan).

### 2.3. RNA Isolation and Quantitative Real-Time PCR

RNA was isolated using TRIzol^®^ Reagent (Invitrogen, Waltham, MA, USA) and total RNA concentrations were measured using the NanoDrop^TM^ One Microvolume UV-Vis Spectrophotometer (Thermo Scientific^TM^, Waltham, MA, USA). Then, 2 μg of total RNA was used to produce complementary DNA (cDNA) using the High-Capacity cDNA Reverse Transcription Kit (Applied Biosystems, Waltham, MA, USA), according to the manufacturer’s protocol. Quantitative real-time PCR (qPCR) was performed by plating three technical replicates per sample and using PerfeCTa SYBR^®^ Green FastMix (Quantabio, Beverly, MA, USA) on the CFX384 Touch^TM^ Real-Time PCR Detection System (Bio-Rad Laboratories, Hercules, CA, USA). The cycling parameters were set to 95 °C for 10 min for polymerase activation, followed by 40 cycles of denaturing at 95 °C for 10 s, annealing at 60 °C for 10 s, and extension at 72 °C for 15 s with a slow-ramp rate of 2.5 °C per second.

We assessed the relative mRNA expression of key genes involved in mitochondrial dynamics (mitochondrial fusion and fission), mitochondrial biogenesis, and mitophagy. Mitofusin 2 (*Mfn2*) mediates outer mitochondrial membrane fusion, while optic atrophy 1 (*Opa1*) mediates inner mitochondrial fusion; together these factors facilitate the tethering and docking of separate, neighboring mitochondria for subsequent unification [41]. Dynamin-related protein 1 (*Drp1*) is a cytoplasmic protein that is integral for mitochondrial fission [41]. Adaptor proteins located on the outer mitochondrial membrane like fission 1 (*Fis1*) recruit DRP1 to the mitochondria where it oligomerizes and drives membrane constriction and subsequent division into two daughter mitochondria [41]. Mitochondrial transcription factor A (*Tfam*) is a master regulator of mitochondrial biogenesis and mitochondrial DNA copy number; it helps stabilize mtDNA during transcription and replication [42,43]. PTEN-induced putative kinase 1 (*Pink1*) is known to facilitate mitophagy, a form of autophagy that removes damaged mitochondria [44]. Adenine nucleotide translocator (*Ant1*) is an important regulator of PINK1-Parkin-mediated mitophagy [17]. Beclin 1 (*Becn1*) is also involved in mitophagy as it facilitates the formation of the autophagosome [45]. Sirtuin 3 (*Sirt3*) is a NAD^+^-dependent protein deacetylase localized in the mitochondria and upstream of the PINK1-Parkin pathway of mitophagy [46]. Levels of gene expression were normalized to the geometric means of reference genes, beta-2 microglobulin (*B2m*) and hypoxanthine phosphoribosyl transferase 1 (*Hprt*), and calculated using the 2^−ΔΔCt^ method [47]. Primer sequences are provided in Table A1.

### 2.4. Mitochondrial Function

The effects of Phe and chlorinated Phe on SIGC mitochondrial function were assessed using commercially available kits that measure levels of cellular reactive oxygen species (ROS), mitochondrial membrane potential, and ATP production. All assays were read using the Synergy^TM^ H1 microplate reader (Agilent Technologies, Inc.). We further assessed mitochondrial biogenesis by measuring mitochondrial DNA copy number.

Cellular ROS: ROS levels were measured using the Cellular Reactive Oxygen Species Detection Assay kit (ab186027, Abcam, Cambridge, UK) (N = 5–8 independent experiments). SIGCs were seeded at 12,000 cells/well in a black, clear-bottom 96-well plate. After allowing the cells to adhere for 24 h, cells were treated with 0 (vehicle, DMSO), 0.01, 0.1, and 1 μM Phe, 9ClPhe, or 9,10Cl_2_Phe made in supplemented media as described above. In the final hour of treatment, ROS Red Working Solution was prepared according to the manufacturer’s protocol and added to each well. Fluorescence was measured at 520 nm/605 nm (excitation/emission) at 24 h.

Mitochondrial Membrane Potential (Δψ_m_): Mitochondrial membrane integrity following exposure to Phe and chlorinated Phe was evaluated using the fluorescent JC-1 Mitochondrial Membrane Potential Assay Kit (ab113850, Abcam), according to the manufacturer’s protocol (N = 5–8 independent experiments). Cells were seeded at 12,000 cells/well in a black, clear-bottom 96-well plate and were allowed to adhere for 24 h. After washing with 1× dilution buffer, cells were incubated with 20 μM JC-1 dye made in 1× dilution buffer for 10 min at 37 °C in the dark. Cells were then washed twice with 1× dilution buffer and then exposed to 0 (vehicle, DMSO), 0.01, 0.1, and 1 μM Phe, 9ClPhe, or 9,10Cl_2_Phe made in 1× dilution buffer supplemented with 10% (*v*/*v*) FBS. After 20 h, untreated control wells were treated with 100 μM carbonyl cyanide 4-(trifluoromethoxy) phenylhydrazone (FCCP) to serve as a positive control, as per the manual’s instructions (see Appendix A.2). After 4 h (t = 24 h), fluorescence was measured at 475 nm excitation and 590 nm (aggregate) or 530 nm (monomer) emission.

ATP production: As the primary function of the mitochondria is to produce energy for cells, ATP levels following exposure to Phe and chlorinated Phe were evaluated using the Luminescent ATP Detection Assay Kit (ab113849, Abcam), according to the manufacturer’s protocol (N = 3–6 independent experiments). Cells were seeded at 12,000 cells/well in a white, clear-bottom 96-well plate and treated with 0 (vehicle, DMSO), 0.01, 0.1, and 1 μM Phe, 9ClPhe, or 9,10Cl_2_Phe made in supplemented media as described above. After 24 h of treatment, luminescence was measured.

mtDNA copy number (mtDNA-CN): SIGCs were seeded and treated with 0 (vehicle, DMSO), 0.01, 0.1, or 1 μM Phe or chlorinated Phe in 6-well plates, as described above. After 24 h, genomic DNA (gDNA) was isolated using the PureLink^TM^ Genomic DNA Mini Kit (Invitrogen, K182002), according to the manufacturer’s protocol. Briefly, cells were washed twice with Dulbecco’s PBS (DPBS, 1× without calcium and magnesium, Corning) and were collected by trypsinization and centrifugation. The cell pellet was resuspended in 200 μL DPBS (1× with calcium and magnesium, Corning) and incubated at room temperature with 20 μL proteinase K and RNase A for 2 min. This mixture was then incubated at 55 °C for 10 min with 200 μL PureLink Genomic Lysis/Binding Buffer to promote protein digestion. To the lysate, 200 μL of 95% ethanol was added and gDNA was isolated, washed, and purified using spin column-based centrifugation at room temperature, according to the protocol. gDNA was eluted with 35 μL nuclease-free H_2_O and total gDNA concentrations were measured using the NanoPhotometer^®^ N60 (Implen, Munich, Germany). gDNA samples were subsequently diluted to 10 ng/μL using nuclease-free H_2_O.

mtDNA copy number was evaluated by qPCR using the same conditions as described above. Specific primers to assess mtDNA-CN are provided in Table A2. Each reaction contained 250 nM of primer, 5 μL of PerfeCTa SYBR^®^ Green FastMix, and 2.5 μL of diluted gDNA (final concentration 25 ng). mtDNA copy number was analyzed using the 2^−ΔΔCT^ method [48,49].

Immunofluorescence: Colocalization of the mitochondrial marker translocase of outer mitochondrial membrane 20 (TOM20) and the autophagy marker microtubule-associated protein 1A/1B-light chain 3 (LC3) has been previously used to assess mitophagy [17]. For immunofluorescent staining, SIGCs seeded in 8-well chamber slides were exposed to vehicle (DMSO) or Phe, 9ClPhe, or 9,10Cl_2_Phe (0.01–1 μM) for 24 h. After treatment, cells were fixed with 100% methanol at room temperature for 20 min, then blocked with 5% goat serum containing 0.3% Triton X-100 for 1 h at 37 °C in a humidified atmosphere. Cells were then incubated with primary antibodies overnight at 4 °C, washed three times with DPBS (5 min/wash), and incubated with secondary antibodies for 1 h at room temperature in the dark. SIGCs were counterstained with ProLong^TM^ Gold Antifade Mountant with DAPI (Cell Signaling Technologies, Danvers, MA, USA) and mounted with coverslips. After 24 h, cells were imaged using the Nikon Eclipse Ni-E microscope equipped with a DS-Qi2 camera (Nikon, Tokyo, Japan). Antibody details are provided in Table A3. Images were analyzed using Fiji [50] and representative images are provided in Appendix A.2.

### 2.5. TUNEL Assay

Apoptosis was measured via TUNEL (terminal deoxynucleotidyl transferase dUTP nick-end labeling) staining using the In Situ Cell Death Detection Kit, Fluorescein (Roche, Basin, Switzerland), according to the manufacturer’s protocol. SIGCs were seeded in 8-well chamber slides and exposed to vehicle (DMSO) or Phe, 9ClPhe, or 9,10Cl_2_Phe (0.01–1 μM) for 24 h. After treatment, cells were fixed with 4% paraformaldehyde for 15 min at room temperature and washed twice with DPBS before permeabilizing with 0.1% Triton X-100 in 0.1% sodium citrate. For the positive control, additional cells treated with vehicle were incubated with DNase I recombinant, RNase-free (Roche) made in 50 mM Tris-HCl, pH 7.5, 10 mM MgCl_2_, and 1 mg/mL BSA for 10 min at room temperature. Samples were washed twice and incubated with fluorescein-labeled TUNEL enzyme for 1 h at 37 °C in a humidified atmosphere. Samples were counterstained in ProLong^TM^ Gold Antifade Mountant with DAPI and mounted in coverslips. Cells were imaged using the Nikon Eclipse Ni-E microscope with a Nikon DS-Qi2 camera. Images were analyzed using Fiji [50]. The percentage of apoptotic cells was calculated as the average of TUNEL-positive nuclei relative to total number of nuclei present in three fields of view per well, for each treatment group per slide. Representative images and quantification of DNase I positive controls are provided in Appendix A.2.

### 2.6. Statistical Analysis

Statistical significance for all data was assessed using GraphPad Prism (v11.0.0, GraphPad Software, Boston, MA, USA). Data outliers were identified using Grubb’s outlier test, and normality was assessed using the Shapiro–Wilk test, then tested for equal variance. Comparisons between control and treatment groups were conducted using a one-way ANOVA. If the ANOVA was significant (*p* < 0.05), post hoc analysis using the Dunnett method was performed. For data that did not pass normality or equal variance testing, data were analyzed using a Kruskal–Wallis test. If the Kruskal–Wallis test was significant (*p* < 0.05), treatment groups were compared to control using Dunn’s post hoc test. Data were presented as mean SEM and were considered significant when *p* < 0.05.

## 3. Results

### 3.1. Phe and Chlorinated Phes Differentially Affect Hormone Production in Granulosa Cells

**Figure 1:**
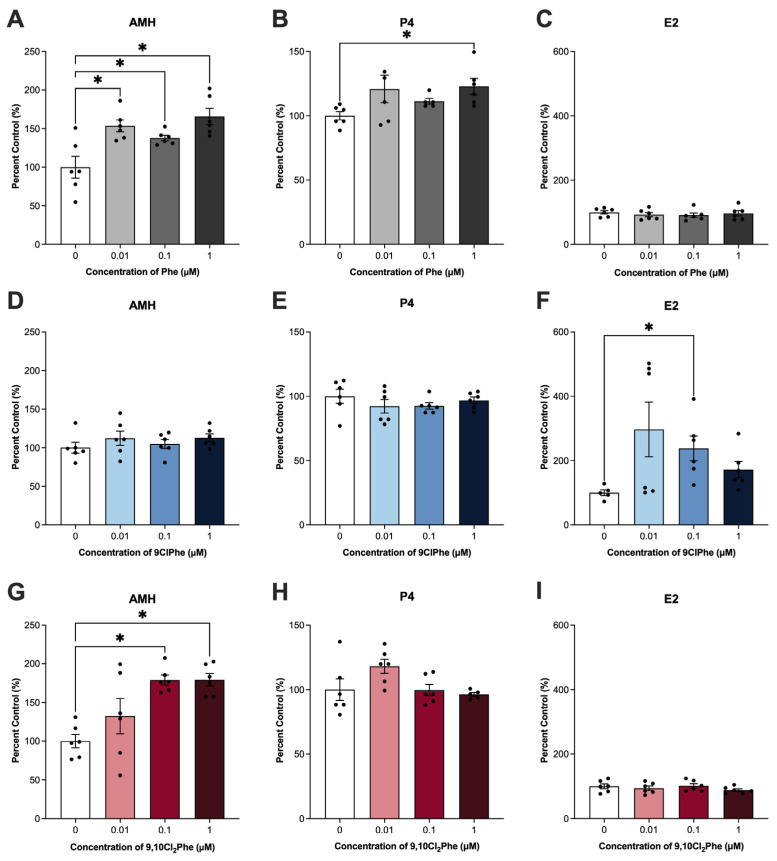
Hormone quantification in spontaneously immortalized granulosa cells (SIGCs) treated with 0 (vehicle, DMSO), 0.01, 0.1, and 1 μM phenanthrene (Phe) (**A**–**C**), 9-chlorophenanthrene (9ClPhe) (**D**–**F**), and 9,10-dichlorophenanthrene (9,10Cl_2_Phe) (**G**–**I**) for 24 h. Anti-Müllerian hormone (AMH), progesterone (P4), and estradiol (E2) data are presented as mean percent control ± SEM (N = 6). Statistical significance compared to control is indicated by asterisks * *p* < 0.05.

In the ovary, AMH, P4, and E2 are major reproductive hormones secreted by granulosa cells. Exposure to both Phe and 9,10Cl_2_Phe significantly increased AMH production, with Phe elevating AMH levels at all doses tested (Figure 1A,G). Interestingly, only Phe altered P4 production, with 1 μM Phe producing a significant increase (Figure 1B). In contrast, only 9ClPhe altered E2 secretion (ANOVA *p* < 0.05), increasing E2 levels to 300%, 240%, and 172% of control at 0.01, 0.1, and 1 μM, respectively; however only 0.1 μM reached statistical significance (*p* = 0.02), whereas 0.01 and 1 μM 9ClPhe did not (*p* = 0.06 and *p* = 0.12, respectively) (Figure 1F).

### 3.2. Phe and Chlorinated Phe Have Opposing Effects on Markers of Mitochondrial Dynamics, but Do Not Affect mtDNA Copy Number

**Figure 2:**
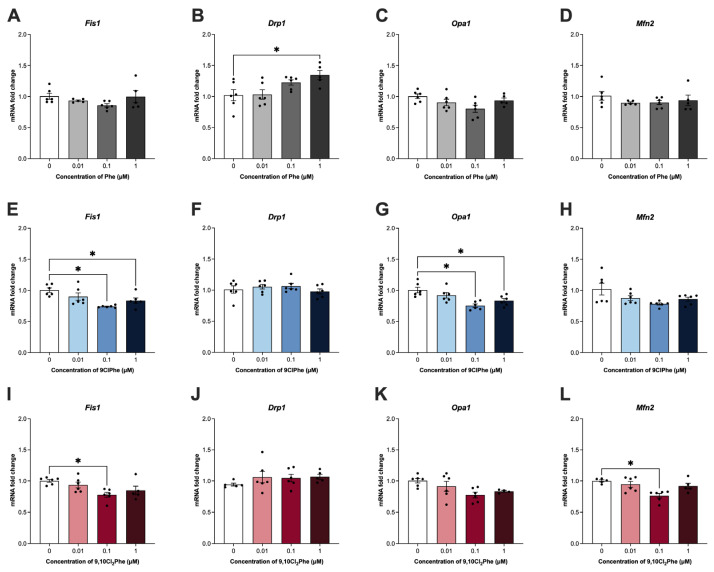
Assessment of mitochondrial dynamics markers in spontaneously immortalized granulosa cells (SIGCs) treated with 0 (vehicle, DMSO), 0.01, 0.1, and 1 μM phenanthrene (Phe) (**A**–**D**), 9-chlorophenanthrene (9ClPhe) (**E**–**H**), and 9,10-dichlorophenanthrene (9,10Cl_2_Phe) (**I**–**L**) for 24 h. Relative mRNA fold change in mitochondrial fission 1 (*Fis1*), dynamin-related protein 1 (*Drp1*), optic atrophy 1 (*Opa1*), and mitofusin 2 (*Mfn2*) expressions are presented as mean ± SEM (N = 6). Statistical significance compared to control is indicated by asterisks * *p* < 0.05.

**Figure 3:**
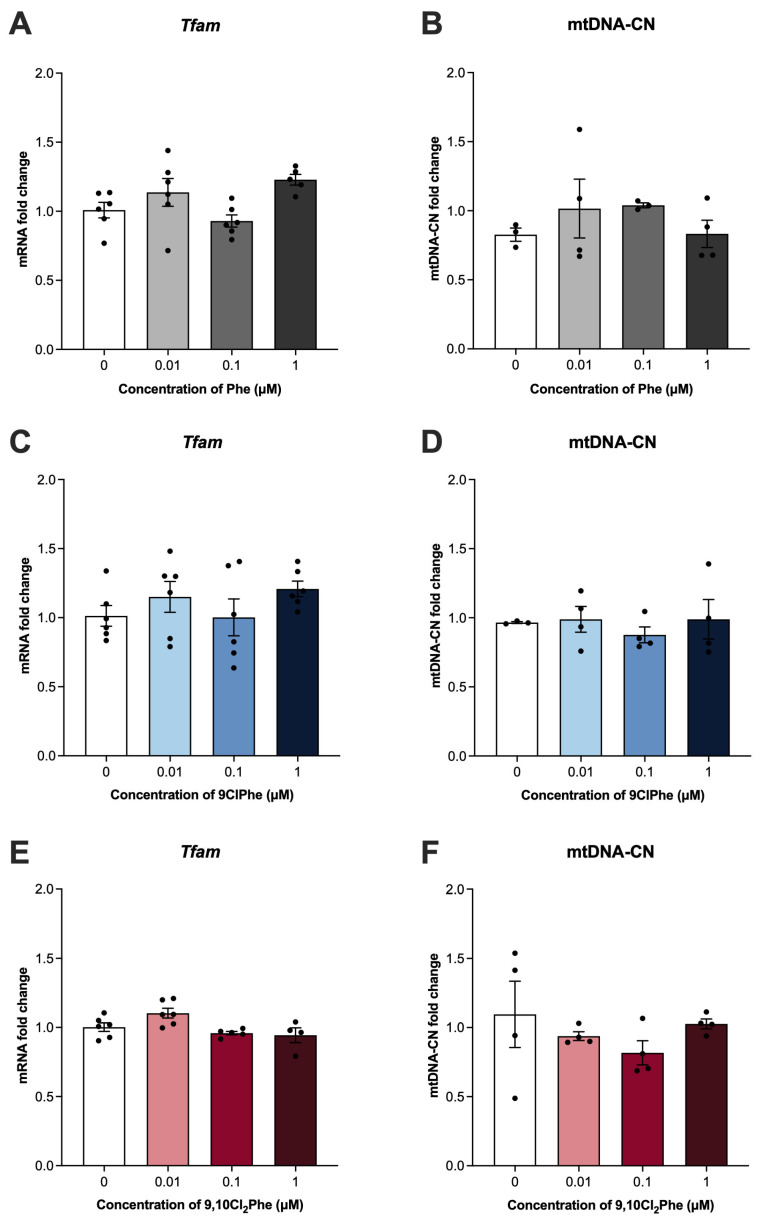
Assessment of mitochondrial biogenesis and mitochondrial DNA copy number (mtDNA-CN) in spontaneously immortalized granulosa cells (SIGCs) treated with 0 (vehicle, DMSO), 0.01, 0.1, and 1 μM phenanthrene (Phe) (**A**,**B**), 9-chlorophenanthrene (9ClPhe) (**C**,**D**), and 9,10-dichlorophenanthrene (9,10Cl_2_Phe) (**E**,**F**) for 24 h. Relative mRNA fold change in mitochondrial transcription factor A (*Tfam*) expression and quantification of mtDNA-CN are presented as mean ± SEM (N = 4–6).

Since steroid hormone synthesis begins at the mitochondria [51], the effects of Phe and its chlorinated congeners on markers of mitochondrial dynamics were examined and appear to be compound specific. Treatment with 1 μM Phe significantly increased the expression of mitochondrial fission marker *Drp1* (Figure 2B) with no significant effect on any other marker of mitochondrial dynamics. In contrast, neither 9ClPhe nor 9,10Cl_2_Phe treatment affected the expression of *Drp1* (Figure 2F,J). 9ClPhe treatment significantly reduced the expression of *Fis1* and *Opa1* (Figure 2E,G), whereas 9,10Cl_2_Phe significantly decreased expression of *Fis1* and *Mfn2* (Figure 2I,L). We further assessed the impact of Phe and the chlorinated Phes on mitochondrial biogenesis and saw no significant differences in *Tfam* mRNA expression or mtDNA-CN following exposure to any of the three compounds (Figure 3).

### 3.3. Chlorinated Phes Reduce Mitochondrial Membrane Potential, but Only 9,10Cl_2_Phe Significantly Increases ROS

**Figure 4:**
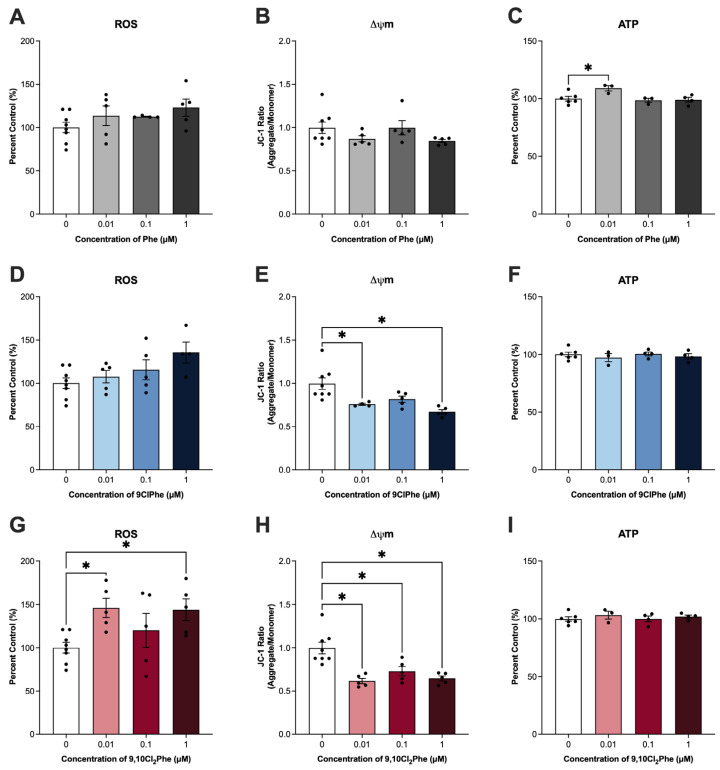
Assessment of mitochondrial function in spontaneously immortalized granulosa cells (SIGCs) treated with 0 (vehicle, DMSO), 0.01, 0.1, and 1 μM phenanthrene (Phe) (**A**–**C**), 9-chlorophenanthrene (9ClPhe) (**D**–**F**), and 9,10-dichlorophenanthrene (9,10Cl_2_Phe) (**G**–**I**) for 24 h. Quantification of reactive oxygen species (ROS) and ATP levels are shown as percent control. Fluorescence intensity ratios of JC-1 aggregates and monomers were quantified to assess mitochondrial membrane potential (Δψ_m_). Data is presented as mean ± SEM (N = 3–8). Statistical significance compared to control is indicated by asterisks * *p* < 0.05.

Disruption in mitochondrial dynamics can impact mitochondrial function, leading to altered mitochondrial membrane potential (Δψ_m_) and ROS and ATP levels, key indicators of mitochondrial activity [52]. Exposure to 9ClPhe and 9,10Cl_2_Phe significantly altered ROS production and Δψ_m_. Specifically, 9ClPhe resulted in a non-significant increase in ROS (ANOVA *p* = 0.07) and significantly reduced Δψ_m_ at 0.01 and 1 μM (Figure 4D,E). A more pronounced effect was observed with 9,10Cl_2_Phe, which significantly increased ROS and decreased Δψ_m_ at both 0.01 and 1 μM (Figure 4G,H). While Phe had no significant effect on ROS or Δψ_m_, Phe alone increased ATP at the 0.01 μM concentration (Figure 4C).

### 3.4. Chlorinated Phe Exposure Reduces the Expression of Key Markers for Mitophagy

**Figure 5:**
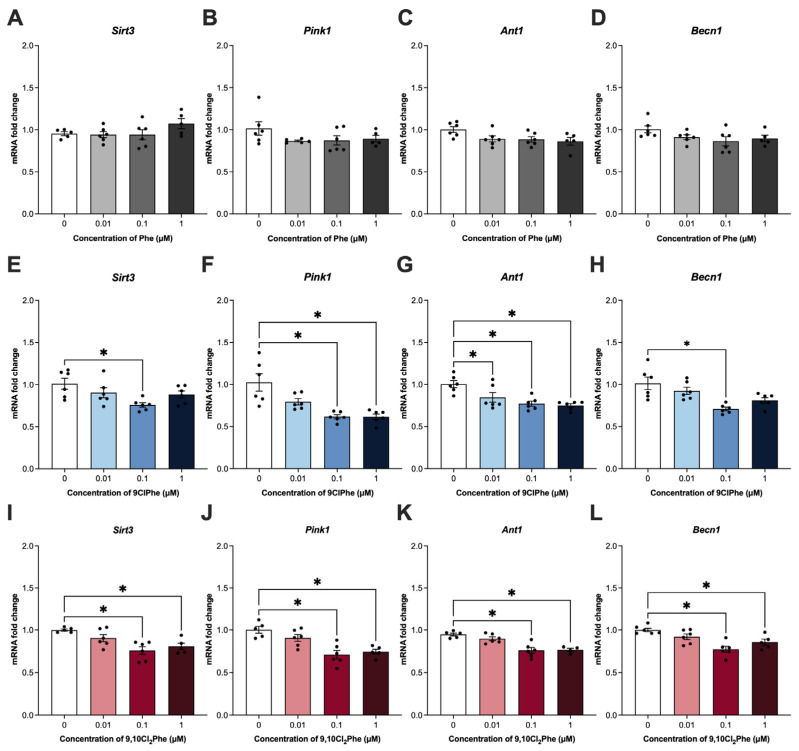
Relative mRNA expression of markers for mitophagy in spontaneously immortalized granulosa cells (SIGCs) treated with 0 (vehicle, DMSO), 0.01, 0.1, and 1 μM phenanthrene (Phe) (**A**–**D**), 9-chlorophenanthrene (9ClPhe) (**E**–**H**), and 9,10-dichlorophenanthrene (9,10Cl_2_Phe) (**I**–**L**) for 24 h. Data for sirtuin 3 (*Sirt3*), PTEN-induced putative kinase 1 (*Pink1*), adenine nucleotide translocator (*Ant1*), and beclin 1 (*Becn1*) are presented as mean ± SEM (N = 6). Statistical significance compared to control is indicated by asterisks * *p* < 0.05.

**Figure 6:**
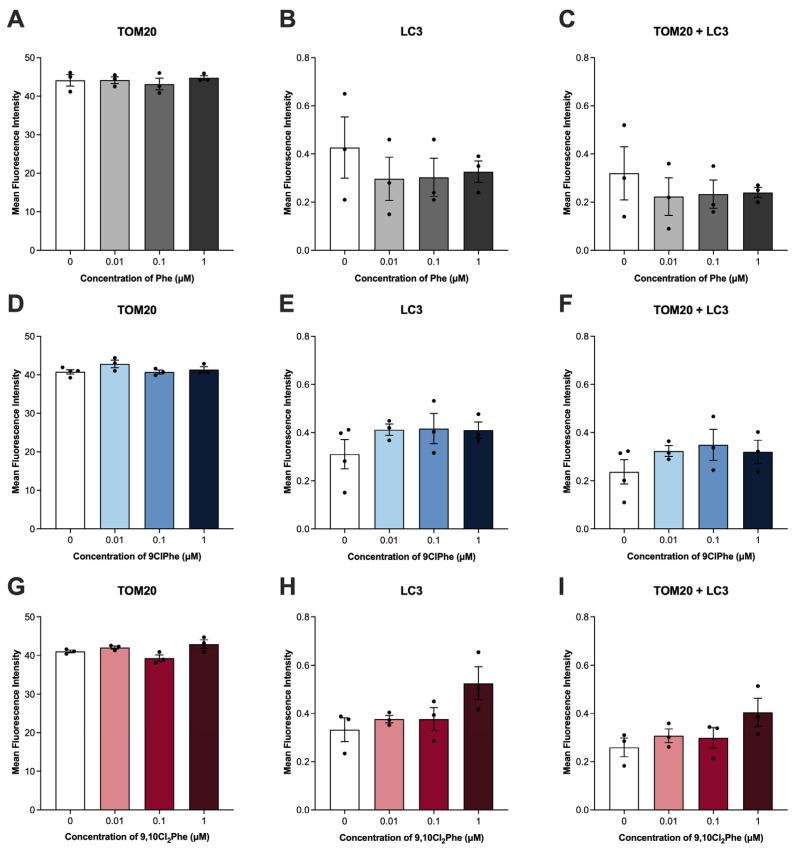
Assessment of mitophagy in spontaneously immortalized granulosa cells (SIGCs) treated with 0 (vehicle, DMSO), 0.01, 0.1, and 1 μM phenanthrene (Phe) (**A**–**C**), 9-chlorophenanthrene (9ClPhe) (**D**–**F**) and 9,10-dichloropheanthrene (9,10Cl_2_Phe) (**G**–**I**) for 24 h by immunofluorescence. Quantification of TOM20 and LC3 and colocalization of TOM20 and LC3 are shown as mean fluorescence intensity ± SEM (N = 3–4).

As a mechanism of mitochondrial quality control, mitophagy refers to the process by which cells selectively target and remove damaged mitochondria [44]. As such, we then examined key markers of mitophagy. Treatment with Phe had no effect on the expression of any of the mitophagy markers tested (Figure 5A–D), consistent with unchanged TOM20 and LC3 colocalization (Figure 6A–C). In contrast, treatment with 0.1 and 1 μM 9,10Cl_2_Phe significantly decreased the expression of all markers of mitophagy (Figure 5I–L). At these concentrations, a slight increase in TOM20-LC3 colocalization was observed; however, this did not reach statistical significance (ANOVA *p* = 0.1) (Figure 6G–I). Similarly, treatment with 0.1 μM 9ClPhe decreased the expression of all mitophagy markers (Figure 5E–H), while no significant change in TOM20-LC3 colocalization was detected (Figure 6D–F).

### 3.5. Phe and Chlorinated Phe Do Not Induce Granulosa Cell Apoptosis

**Figure 7:**
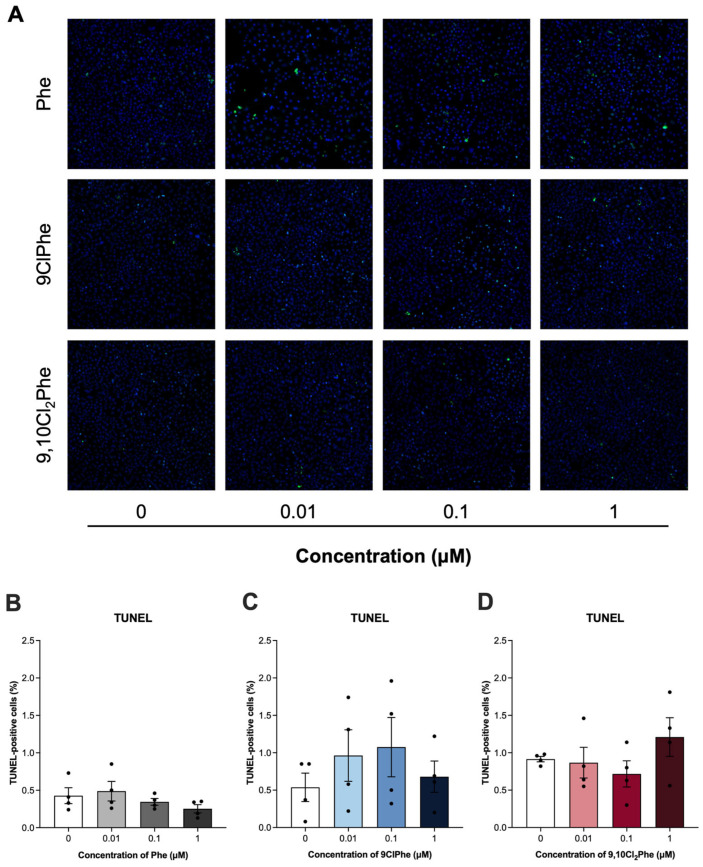
Detection of apoptosis in spontaneously immortalized granulosa cells (SIGCs) treated with 0 (vehicle, DMSO), 0.01, 0.1, and 1 μM phenanthrene (Phe), 9-chlorophenanthrene (9ClPhe), and 9,10-dichlorophenanthrene (9,10Cl_2_Phe) for 24 h via TUNEL staining. Representative images of TUNEL-positive cells (green) and nuclei stained with DAPI (blue) are shown (**A**). The mean percentage of TUNEL-positive cells ± SEM for each chemical is shown (**B**–**D**) (N = 4).

To determine whether changes in mitochondrial function led to cell death, we measured apoptosis using a TUNEL assay. Treatment with Phe had no effect on granulosa cell apoptosis. Similarly, neither chlorinated Phe congener significantly increased apoptosis at the concentrations tested despite their effects on mitochondrial function and mitophagy-related pathways (Figure 7).

## 4. Discussion

To date, our understanding of the toxic effects and health risks posed by exposure to halogenated PAHs are limited despite their prevalence in various matrices [53,54]. Although several studies have reported the endocrine-disruptive potential and reproductive toxicity of Phe [5,8,55], whether chlorination influences these effects has not been investigated. Previous studies, including work from our group, have shown that hormone production following exposure to parent PAHs can differ from that observed with their alkylated analogs [56,57], suggesting that structural modifications may alter endocrine outcomes. Given this, the present study aimed to compare and characterize the effects of Phe and chlorinated Phe congeners on hormone production in granulosa cells.

We observed that Phe significantly increased both AMH and P4 levels, whereas 9,10Cl_2_Phe elevated AMH levels only. In contrast to Phe and 9,10Cl_2_Phe, 9ClPhe had no effect on AMH or P4 and selectively increased E2 production. Several studies have demonstrated that exposure to PAHs can modulate E2 levels, although the direction and magnitude of these effects vary across PAHs, models, and exposure methods [56,57,58,59,60,61]. AMH, a key regulator of follicle development secreted by granulosa cells of preantral and antral follicles, is often elevated in ovarian disorders such as PCOS [62,63]. In our study, both Phe and 9,10Cl_2_Phe increased AMH secretion by granulosa cells; this increase in AMH secretion is a clinical feature observed in patients with PCOS [63]. Epidemiological studies in Chinese women report that total serum PAH levels (including naphthalene, acenaphthylene, acenaphthene, fluorene, and phenanthrene) were positively associated with PCOS risk [64]. Additionally, luteinizing hormone (LH), which positively regulates AMH, was elevated in patients with PCOS and positively correlated with both serum PAH and urinary hydroxylated PAH levels [64,65]. Notably, these studies found no significant difference in E2 levels between PCOS and control groups [64,65], consistent with our finding that Phe and 9,10Cl_2_Phe increased AMH without altering E2 output in SIGCs. The current study suggests that the association between altered AMH levels and Phe in clinical populations may be a direct effect of Phe at the level of the ovarian granulosa cells. Moreover, these results indicate that Phe and its chlorinated congeners differentially alter hormone production in granulosa cells and that all of these compounds may function as endocrine-disrupting chemicals with unique effects.

Since mitochondria are the initial and rate-limiting sites of steroidogenesis [51], we further examined how Phe and its chlorinated congeners affected mitochondrial function. Exposure to 1 μM Phe significantly increased mRNA expression of mitochondrial fission marker *Drp1*, suggesting that Phe enhances mitochondrial fragmentation [66]. This is consistent with studies in rats exposed to BaP or its metabolite, benzo[*a*]pyrene-7,8-dihydrodiol-9,10-epoxide (BPDE), which reported increased DRP1 protein expression, along with swollen and disorganized mitochondria and reduced mitochondrial membrane potential [14,67]. Similarly, mouse oocytes exposed to 400 μM Phe demonstrated significant increases in expression of mitochondrial fission markers *Fis1* and *Drp1*, and decreased expression of mitochondrial fusion marker *Mfn2* [55], which were accompanied by reduced mitochondrial membrane potential and increased intracellular calcium, ROS production, DNA damage, and upregulation of pro-apoptotic markers [55]. Interestingly, we did not observe any significant changes in ROS levels or mitochondrial membrane potential following exposure to Phe; however, ATP output was significantly increased at the 0.01 μM dose. This outcome was unexpected as PAH exposure has been linked to mitochondrial dysfunction and reduced ATP production [14]. However, other studies investigating cigarette smoke exposure, which is a mixture known to contain PAHs, have reported altered mitochondrial dynamics alongside increased ATP [68].

Contrastingly, both 9ClPhe and 9,10Cl_2_Phe reduced the expression of mitochondrial fission (*Fis1*) and fusion (*Opa1*, *Mfn2*) markers, suggesting that chlorinated derivatives broadly compromise mitochondrial remodeling and differentially alter mitochondrial structure compared to its parent counterpart. Other studies in ovarian cells have shown that reduced *Mfn2* or *Opa1* expression can also lead to altered mitochondrial structure, loss of mitochondrial membrane potential, reduced ATP production, and increased apoptosis, which may lead to impaired follicle development and oocyte quality [55,69,70,71]. In this study, SIGCs treated with 9ClPhe (ANOVA *p* = 0.07) and 9,10Cl_2_Phe (ANOVA *p* = 0.028) had increased ROS production and significantly decreased mitochondrial membrane potential, suggesting that chlorination of Phe targets the mitochondria without any significant changes on ATP output at 24 h. In a study by Luo et al., both BaP and chlorinated BaP (6-ClBaP) increased ROS and reduced ATP and cell viability of hepatic L02 cells, with 6-ClBaP having more pronounced effects [37]. Despite similar disruptions in mitochondrial function, transcriptomic and metabolomic analyses revealed distinct mechanisms of action where BaP caused transcriptional downregulation of genes encoding the electron transport chain via AhR-mediated signaling, whereas 6-ClBaP caused stronger inhibition of enzymatic activities within the electron transport chain complexes [37]. In line with this, another study by the same group reported that chlorination of pyrene displayed distinct effects on energy production where exposure to the parent compound, pyrene, upregulated fatty acid oxidation and oxidative phosphorylation, enhancing energy production, whereas its chlorinated derivative, 1-chloropyrene, showed both reduced and enhanced energy production depending on the concentration tested [72]. Since a balance of fission and fusion are required to maintain mitochondrial distribution within the ovary, disruption of these processes can adversely affect mitochondrial quality control and impair oocyte quality and fertility [73,74].

In response to stress and damage, mitochondria can replicate their mtDNA and also undergo mitochondrial biogenesis to maintain mitochondrial number and support cellular energy demands [75]. The literature regarding the impact of PAHs on mtDNA-CN is inconsistent, with some studies reporting that PAH exposure increases mtDNA-CN [76], while others report a decrease in mtDNA-CN [77,78]. In our study, we did not observe any significant changes in mtDNA-CN of SIGCs exposed to Phe or its chlorinated congeners. This effect aligns with unchanged expression of *Tfam*, which is a known regulator of mtDNA transcription and replication, and positively correlates with mtDNA abundance [79,80]. Despite the lack of significant changes in mtDNA-CN, disruption in processes such as mitochondrial dynamics, mitochondrial biogenesis, or mitophagy can influence cell fate, particularly influencing mitochondrial quality control and cell death pathways [66]. In fact, ovary homogenates from smoke-exposed mice demonstrated decreased expression of fusion markers, including MFN2, and induction of autophagy [81].

Mitophagy is a selective form of autophagy in which autophagosomes are recruited to damaged or dysfunctional mitochondria for degradation [82]. Exposure to PAHs like BaP has previously been shown to impair mitophagy in ovarian corpora lutea and the KGN human ovarian granulosa cell line by disrupting ANT1-PINK1-Parkin signaling [17]. Consistent with this, we found that 9ClPhe and 9,10Cl_2_Phe significantly decreased the expression of several mitophagy genes, including *Ant1*, *Pink1*, and *Becn1*, whereas Phe had no effect. Both chlorinated congeners also significantly reduced mRNA expression of *Sirt3*, a mitochondrial NAD^+^-dependent deacetylase that regulates mitochondrial functions including mitophagy, redox homeostasis, and mitochondrial dynamics [83]. Since *Sirt3* deletion impairs mitochondrial integrity and follicle development in the ovary [84], decreased *Sirt3* expression following exposure to chlorinated Phe may indicate disruption in mitochondrial quality control in granulosa cells.

To further assess mitophagy, we examined the colocalization of mitochondrial marker TOM20 and autophagosome marker LC3. Despite transcriptional suppression of mitophagy markers after exposure to chlorinated Phe, neither chemical significantly altered TOM20-LC3 colocalization at 24 h. Exposure to 9,10Cl_2_Phe led to a slight, non-significant increase in LC3 and TOM20-LC3 colocalization, which may be explained, in part, by the observed increase in AMH output. AMH has been reported to inhibit FOXO3a, an important regulator upstream of PINK1/Parkin-mediated mitophagy [85]. Given that 9,10Cl_2_Phe increased AMH, it is plausible that FOXO3a-dependent signaling required for autophagosome recruitment is suppressed and prevents removal of damaged mitochondria despite observed transcriptional changes. Alternatively, these results may also indicate that transcriptional suppression of mitophagy markers precedes detectable changes in autophagosome recruitment and that the removal of damaged mitochondria occurs at timepoints other than 24 h.

Exposure to Phe or its chlorinated derivatives had no significant effect on granulosa cell apoptosis. This contrasts findings reported by Cai et al. (2024), where mono- and di-chlorinated naphthalenes (PCN) had differential effects on cytotoxicity and 2-chloronapthalene increased both autophagy and apoptosis in MCF-10A cells [86]. These distinctions may reflect differences in the specific PAHs tested, exposure concentrations and durations, or cell-type specific responses. Since oxidative stress and mitophagy inhibition are early events of granulosa cell damage, long-term exposures may trigger apoptosis or other forms of programmed cell death. Overall, this study shows that chlorinated congeners of Phe can induce mitochondrial dysfunction in granulosa cells without causing apoptosis at 24 h and suggests that the observed disruptions in ROS production, mitochondrial membrane potential, and mitophagy-related pathways are early stress responses to chlorinated Phe exposure.

Given the critical role of mitochondria in granulosa cell steroidogenesis, mitochondrial perturbations induced by chlorinated Phe may contribute to altered hormone production and endocrine disruption, potentially affecting ovarian function and female fertility. It is important to emphasize that the results of this study focused on short-term exposures (24 h) and are specific to SIGCs, an immortalized rat granulosa cell line that has been used as a model for steroidogenesis to represent granulosa cells of developing follicles since they do not undergo luteinization [87]. Future studies should explore long-term exposures and confirm whether Phe and ClPhe elicit similar effects in other mammalian culture models (i.e., rat, mouse, human) including primary granulosa cells, isolated follicles, or whole ovarian cultures, which can capture *in vivo* hormonal responsiveness, follicle development, and ovarian complexity. Moreover, the biotransformation of PAHs can generate metabolites with distinct toxicity profiles from their parent compound, a consideration not explored in this study. Future *in vivo* studies which incorporate metabolic processes are needed to confirm the contribution of Phe and ClPhe to ovarian toxicity.

## 5. Conclusions

This study provides molecular insight into the effects of Phe and its chlorinated congeners, 9ClPhe and 9,10Cl_2_Phe, on hormone production, mitochondrial function, and cell death pathways in mammalian granulosa cells. To our knowledge, this is the first study to assess the endocrine effects of chlorinated derivatives of Phe. Future studies should investigate the specific molecular mechanisms underlying Phe- and chlorinated Phe-mediated alterations in steroidogenesis and mitochondrial function in granulosa cells (i.e., AhR or estrogen receptor signaling pathways). Continued efforts to monitor the detection of ClPhe and other halogenated PAHs in biological tissues are needed to better understand exposure, and subsequent research should evaluate the consequences of long-term exposure on steroidogenesis, follicle development, and fertility in animal and human populations. Given the growing detection and widespread prevalence of halogenated PAHs in the environment, these findings highlight the importance of evaluating broader classes of PAHs for their potential impacts on ovarian function and female reproductive health.

## Figures and Tables

**Figure A1:**
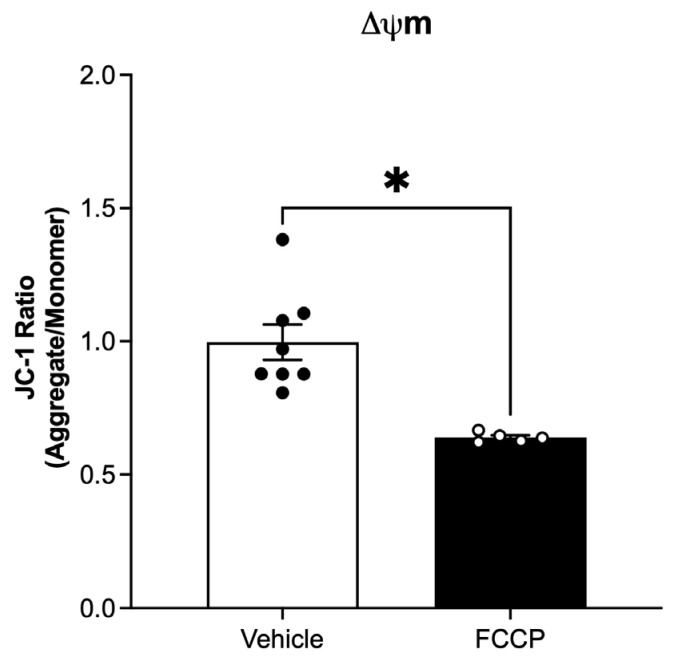
Assessment of mitochondrial membrane potential in spontaneously immortalized granulosa cells (SIGCs) treated with vehicle (DMSO) or 100 μM carbonyl cyanide 4-(trifluoromethoxy) phenylhydrazone (FCCP) for 4 h. Fluorescence intensity ratios of JC-1 aggregates and monomers were quantified to assess mitochondrial membrane potential (Δψ_m_). Data is presented as mean ± SEM (N = 3–8). Comparisons to control were determined using the Mann-Whitney test, and statistical significance is indicated by asterisks * *p* < 0.05.

**Figure A2:**
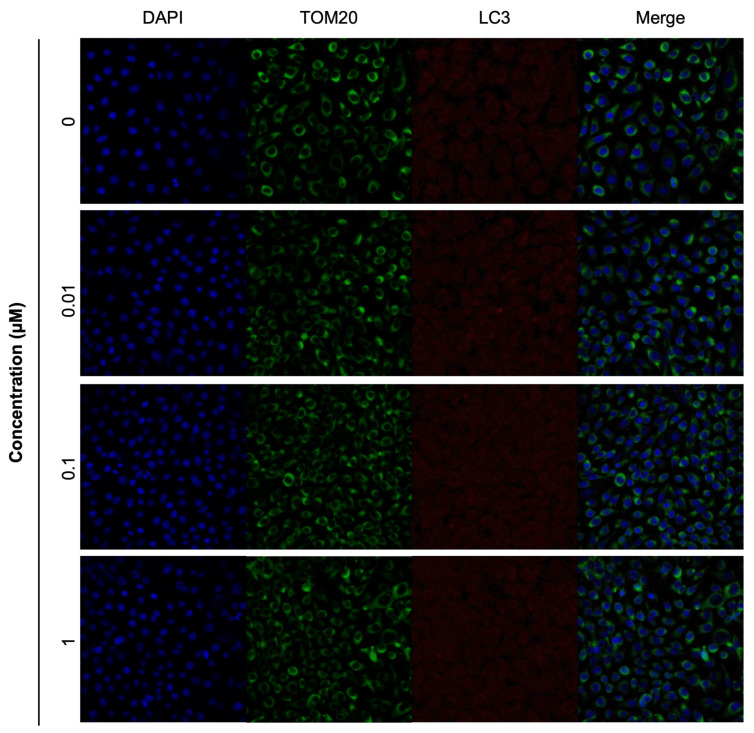
Colocalization of mitochondrial marker TOM20 and autophagy marker LC3 in spontaneously immortalized granulosa cells (SIGCs) treated with 0 (vehicle, DMSO), 0.01, 0.1, and 1 μM phenanthrene (Phe) for 24 h by immunofluorescence. Representative images show nuclei (DAPI, blue), TOM20 (green), LC3 (red), and merged (nuclei, TOM20, and LC3).

**Figure A3:**
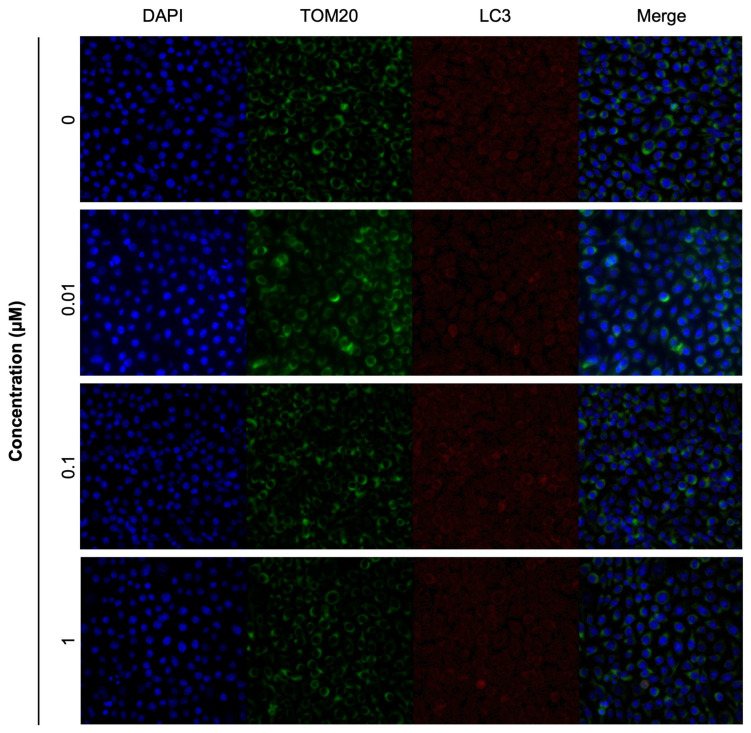
Colocalization of mitochondrial marker TOM20 and autophagy marker LC3 in spontaneously immortalized granulosa cells (SIGCs) treated with 0 (vehicle, DMSO), 0.01, 0.1, and 1 μM 9-chlorophenanthrene (9ClPhe) for 24 h by immunofluorescence. Representative images show nuclei (DAPI, blue), TOM20 (green), LC3 (red), and merged (nuclei, TOM20, and LC3).

**Figure A4:**
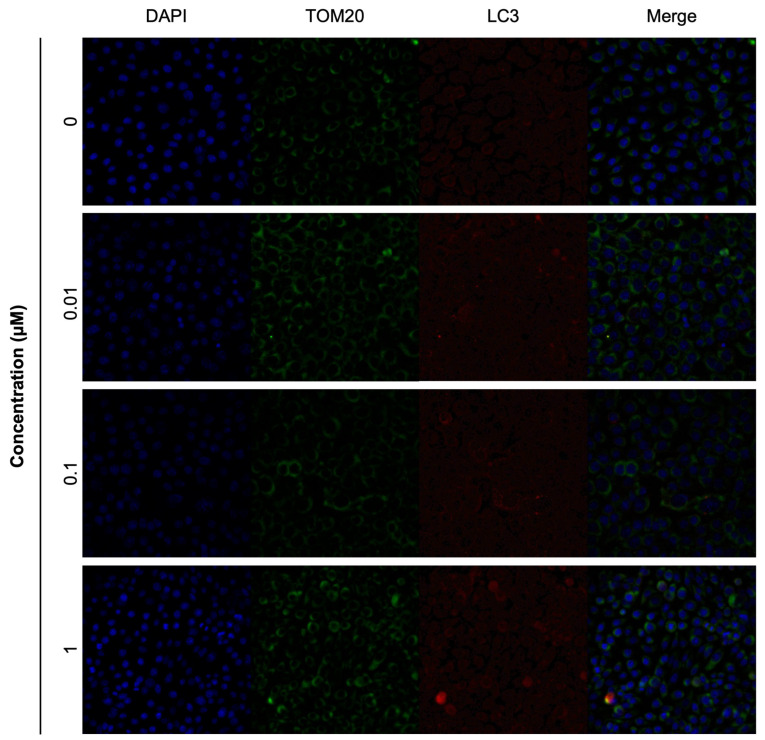
Colocalization of mitochondrial marker TOM20 and autophagy marker LC3 in spontaneously immortalized granulosa cells (SIGCs) treated with 0 (vehicle, DMSO), 0.01, 0.1, and 1 μM 9,10-dichlorophenanthrene (9,10Cl_2_Phe) for 24 h by immunofluorescence. Representative images show nuclei (DAPI, blue), TOM20 (green), LC3 (red), and merged (nuclei, TOM20, and LC3).

**Figure A5:**
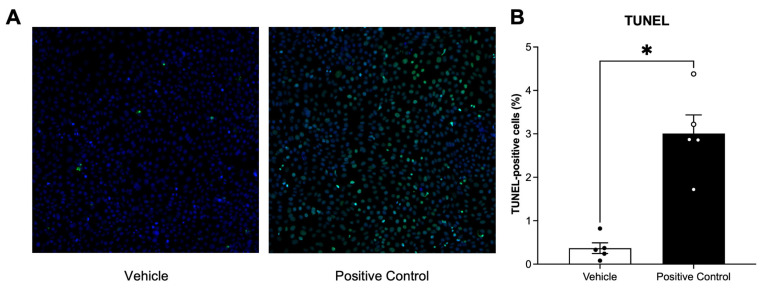
Detection of apoptosis in spontaneously immortalized granulosa cells (SIGCs) treated with a vehicle (DMSO) or positive control (DNase I) via TUNEL staining. Representative images of TUNEL-positive cells (green) and nuclei stained with DAPI (blue) are shown (**A**). The mean percentage of TUNEL-positive cells ± SEM is shown (**B**) (N = 4). Comparisons to control were determined using the Mann-Whitney test, and statistical significance is indicated by asterisks * *p* < 0.05.

## Data Availability

The original contributions presented in this study are included in the article. Further inquiries can be directed to the corresponding author.

## Abbreviations

The following abbreviations are used in this manuscript:

PAHs	Polycyclic aromatic hydrocarbons
ClPAHs	Chlorinated polycyclic aromatic hydrocarbons
Phe	Phenanthrene
ClPhe	Chlorinated phenanthrenes
9ClPhe	9-chlorophenanthrene
9,10Cl_2_Phe	9,10-dichlorophenanthrene
PCOS	Polycystic ovary syndrome
POI	Premature ovarian insufficiency
SIGCs	Spontaneously immortalized granulosa cells
AMH	Anti-Müllerian hormone
P4	Progesterone
E2	Estradiol
Mfn2	Mitofusin 2
Opa1	Optic atrophy 1
Drp1	Dynamin-related protein 1
Fis1	Fission 1
Tfam	Mitochondrial transcription factor A
Pink1	PTEN-induced putative kinase 1
Ant1	Adenine nucleotide translocator
Becn1	Beclin 1
Sirt3	Sirtuin 3
B2m	Beta-2 microglobulin
Hprt	Hypoxanthine phosphoribosyl transferase 1
TOM20	Translocase of outer mitochondrial membrane 20
LC3	Microtubule-associated protein 1A/1B-light chain 3
ROS	Reactive oxygen species
ATP	Adenine triphosphate
Δψ_m_	Mitochondrial membrane potential
mtDNA-CN	Mitochondrial DNA copy number
gDNA	Genomic DNA
TUNEL	Terminal deoxynucleotidyl transferase dUTP nick-end labeling
LH	Luteinizing hormone
BaP	Benzo[*a*]pyrene
BPDE	Benzo[*a*]pyrene-7,8-dihydrodiol-9,10-epoxide
FOXO3a	Forkhead box O3
PCN	Polychlorinated naphthalene
NAD^+^	Nicotinamide adenine dinucleotide
FCCP	Carbonyl cyanide 4-(trifluoromethoxy) phenylhydrazone

## Appendix A

### Appendix A.1

**Table A1:** Primer sequences for quantitative real-time PCR (qPCR).

Gene Name	Gene Symbol	Forward Sequence (5′-3′)	Reverse Sequence (5′-3′)	Accession Number
Beta-2 microglobulin	*B2m*	AATTCACACCCACCGAGACC	GCTCCTTCAGAGTGACGTGT	NM_012512.2
Hypoxanthine phosphoribosyl transferase 1	*Hprt*	GCAGTACAGCCCCAAAATGG	GGTCCTTTTCACCAGCAAGCT	NM_012583.2
Fission 1	*Fis1*	CGTGCTTTCTGTAACGCCTG	GCAACCCTGCAATCCTTCAC	NM_001105919.2
Dynamin-related protein 1	*Drp1*	AGTGCTGGAAAGCCTAGTGG	CAACTCCATTTTCTTCTGTTGT	NM_053655.3
Optic atrophy 1	*Opa1*	CCAATTTCGCTCCTGACCTG	GGTGTACCCGCAGTGAAGAA	NM_133585.3
Mitofusin 2	*Mfn2*	TGAATCGGCACAGAGGAGAC	TCTTGAACCTGACGATGGA	NM_130894.4
Mitochondrial transcription factor A	*Tfam*	AATGTGGGGCGTGCTAAGAA	ACAGATAAGGCTGACAGGCG	NM_031326.2
Sirtuin 3	*Sirt3*	AAGCCCTTTTTCACGTTGGC	CTGTGTGTAGAGCCGCAGAA	NM_001106313.2
Adenine nucleotide translocator 1	*Ant1*	TGTACACGGGGACAGTTGAC	CGTTGGACCAAGCACCTTTG	NM_053515.1
Putative kinase 1	*Pink1*	AATGCCGCTGTGTATGAAGC	TCTGCTCCCTTTGAGACGAC	NM_001106694.1
Beclin 1	*Becn1*	GGTCCTGAGGTGAAGAGCG	CAGTTTCAGAGGCTGGCTACA	NM_053739.2

**Table A2:** Primer sequences for mitochondrial-DNA copy number (mtDNA-CN).

Gene Name	Gene Symbol	Forward Sequence (5′-3′)	Reverse Sequence (5′-3′)
nuclear beta-microglobulin	nt-b2m	TACCCGAGGACGTTCTCCAT	CGTGTGCAAAAACCCTCCTG
mitochondrial cytochrome b	mt-cytb	CATTCATTATCGCCGCCCTTGC	TGGGTCTCCTAGTAGGTCTGG

**Table A3:** Antibodies used for immunofluorescence.

Antibody	Host Species	Dilution	Company	Catalog Number
LC3 (E5Q2K)	Mouse	1:100	Cell Signaling Technologies	83506
TOM20 (D8T4N)	Rabbit	1:100	Cell Signaling Technologies	42406
Anti-Rabbit IgG (H+L) Highly Cross-Adsorbed Secondary, Alexa Fluor^TM^ Plus 555	Goat	1:500	Invitrogen	A32732
Anti-Mouse IgG (H+L) Highly Cross-Adsorbed Secondary, Alexa Fluor^TM^ Plus 647	Goat	1:500	Invitrogen	A32728TR

**Table A4:** Quantification of mean immunofluorescence intensity of mitophagy markers TOM20 and LC3 and their colocalization, presented as mean fluorescence intensity per group (N = 3–4).

	0	0.01	0.1	1	ANOVA *p*-Value
Phenanthrene (Phe)					
TOM20	44.11	44.17	43.15	44.77	0.811
LC3	0.43	0.30	0.30	0.33	0.726
TOM20 + LC3	0.32	0.22	0.23	0.24	0.786
9-Chlorophenanthrene (9ClPhe)					
TOM20	40.79	42.80	40.73	41.34	0.265
LC3	0.31	0.41	0.42	0.41	0.389
TOM20 + LC3	0.24	0.32	0.35	0.32	0.409
9,10-Dichlorophenanthrene (9,10Cl_2_Phe)					
TOM20	41.01	42.04	39.31	42.92	0.04 *
LC3	0.33	0.38	0.38	0.53	0.100
TOM20 + LC3	0.26	0.31	0.30	0.40	0.190

* Post hoc test revealed no significant difference between groups.

**Table A5:** Quantification of TUNEL-positive nuclei, presented as mean percentage of total nuclei per group (N = 3–4).

	0	0.01	0.1	1	ANOVA *p*-Value
Phenanthrene	0.43	0.49	0.34	0.25	0.338
9-Chlorophenanthrene	0.54	0.96	1.08	0.68	0.574
9,10-Dichlorophenanthrene	0.91	0.87	0.72	1.21	0.349

## References

[1] Achten C., Andersson J.T. (2015). Overview of Polycyclic Aromatic Compounds (PAC). Polycycl. Aromat. Compd..

[2] Reizer E., Viskolcz B., Fiser B. (2022). Formation and Growth Mechanisms of Polycyclic Aromatic Hydrocarbons: A Mini-Review. Chemosphere.

[3] Mallah M.A., Changxing L., Mallah M.A., Noreen S., Liu Y., Saeed M., Xi H., Ahmed B., Feng F., Mirjat A.A. (2022). Polycyclic Aromatic Hydrocarbon and Its Effects on Human Health: An Overeview. Chemosphere.

[4] Andersson J.T., Achten C. (2015). Time to Say Goodbye to the 16 EPA PAHs? Toward an Up-to-Date Use of PACs for Environmental Purposes. Polycycl. Aromat. Compd..

[5] Peng X., Sun X., Yu M., Fu W., Chen H., Chen J. (2019). Chronic Exposure to Environmental Concentrations of Phenanthrene Impairs Zebrafish Reproduction. Ecotoxicol. Environ. Saf..

[6] Dai C., Han Y., Duan Y., Lai X., Fu R., Liu S., Leong K.H., Tu Y., Zhou L. (2022). Review on the Contamination and Remediation of Polycyclic Aromatic Hydrocarbons (PAHs) in Coastal Soil and Sediments. Environ. Res..

[7] de Pinho J.V., Rodrigues P.d.A., Guimarães I.D.L., Monteiro F.C., Ferrari R.G., Hauser-Davis R.A., Conte-Junior C.A. (2022). The Role of the Ecotoxicology Applied to Seafood as a Tool for Human Health Risk Assessments Concerning Polycyclic Aromatic Hydrocarbons. Int. J. Environ. Res. Public Health.

[8] Chen Y., Zhang Y., Yu Z., Guan Y., Chen R., Wang C. (2021). Early-Life Phenanthrene Exposure Inhibits Reproductive Ability in Adult Zebrafish and the Mechanism of Action. Chemosphere.

[9] Guo J., Guo W., Zhang T., Zheng Y., Han B., Zhang Z., Liang N., Li Y., Shi Y., Zhang X. (2022). Gestational Exposure to Phenanthrene Induces Follicular Atresia and Endocrine Dyscrasia in F1 Adult Female. Ecotoxicol. Environ. Saf..

[10] Loughery J.R., Kidd K.A., Mercer A., Martyniuk C.J. (2018). Part B: Morphometric and Transcriptomic Responses to Sub-Chronic Exposure to the Polycyclic Aromatic Hydrocarbon Phenanthrene in the Fathead Minnow (*Pimephales promelas*). Aquat. Toxicol..

[11] Zheng Y., Li Y., Yue Z., Samreen, Li Z., Li X., Wang J. (2020). Teratogenic Effects of Environmentally Relevant Concentrations of Phenanthrene on the Early Development of Marine Medaka (*Oryzia melastigma*). Chemosphere.

[12] Zhang Y., Dong S., Wang H., Tao S., Kiyama R. (2016). Biological Impact of Environmental Polycyclic Aromatic Hydrocarbons (ePAHs) as Endocrine Disruptors. Environ. Pollut..

[13] Pan J., Liu P., Yu X., Zhang Z., Liu J. (2024). The Adverse Role of Endocrine Disrupting Chemicals in the Reproductive System. Front. Endocrinol..

[14] Yang J., Xu H., Gao R., Liu X., He J., Zhou M., Ding Y., Li F., Geng Y., Mu X. (2022). Exposure to Benzo(a)Pyrene Damages Mitochondrial Function via Suppressing Mitochondrial Melatonin Receptors in Ovarian Corpus Luteum during Early Pregnancy. Chem.-Biol. Interact..

[15] Siemers K.M., Klein A.K., Baack M.L. (2023). Mitochondrial Dysfunction in PCOS: Insights into Reproductive Organ Pathophysiology. Int. J. Mol. Sci..

[16] Varughese R., Rahman S. (2025). Endocrine Dysfunction in Primary Mitochondrial Diseases. Endocr. Rev..

[17] Li N., Xu H., Liu X., Gao R., He J., Ding Y., Li F., Geng Y., Mu X., Chen X. (2022). Exposure to Benzo(a)Pyrene Suppresses Mitophagy via ANT1-PINK1-Parkin Pathway in Ovarian Corpus Luteum during Early Pregnancy. Sci. Total Environ..

[18] Miao Y., Zhou C., Bai Q., Cui Z., ShiYang X., Lu Y., Zhang M., Dai X., Xiong B. (2018). The Protective Role of Melatonin in Porcine Oocyte Meiotic Failure Caused by the Exposure to Benzo(a)Pyrene. Hum. Reprod..

[19] Roubicek D.A., de Souza-Pinto N.C. (2017). Mitochondria and Mitochondrial DNA as Relevant Targets for Environmental Contaminants. Toxicology.

[20] Ohura T., Sawada K., Amagai T., Shinomiya M. (2009). Discovery of Novel Halogenated Polycyclic Aromatic Hydrocarbons in Urban Particulate Matters: Occurrence, Photostability, and AhR Activity. Environ. Sci. Technol..

[21] Ma J., Horii Y., Cheng J., Wang W., Wu Q., Ohura T., Kannan K. (2009). Chlorinated and Parent Polycyclic Aromatic Hydrocarbons in Environmental Samples from an Electronic Waste Recycling Facility and a Chemical Industrial Complex in China. Environ. Sci. Technol..

[22] Nishimura C., Horii Y., Tanaka S., Asante K.A., Ballesteros F., Viet P.H., Itai T., Takigami H., Tanabe S., Fujimori T. (2017). Occurrence, Profiles, and Toxic Equivalents of Chlorinated and Brominated Polycyclic Aromatic Hydrocarbons in E-Waste Open Burning Soils. Environ. Pollut..

[23] Wang Y., Liao R., Liu W., Kannan K., Ohura T., Wu M., Ma J. (2017). Chlorinated Polycyclic Aromatic Hydrocarbons in Surface Sediment from Maowei Sea, Guangxi, China: Occurrence, Distribution, and Source Apportionment. Environ. Sci. Pollut. Res..

[24] Horii Y., Ohura T., Yamashita N., Kannan K. (2009). Chlorinated Polycyclic Aromatic Hydrocarbons in Sediments from Industrial Areas in Japan and the United States. Arch. Environ. Contam. Toxicol..

[25] Zhao C., Li A., Zhang G., Pan Y., Meng L., Yang R., Li Y., Zhang Q., Jiang G. (2022). Parent and Halogenated Polycyclic Aromatic Hydrocarbons in the Serum of Coal-Fired Power Plant Workers: Levels, Sex Differences, Accumulation Trends, and Risks. Environ. Sci. Technol..

[26] Menzie C.A., Potocki B.B., Santodonato J. (1992). Exposure to Carcinogenic PAHs in the Environment. Environ. Sci. Technol..

[27] Wang L., Li C., Jiao B., Li Q., Su H., Wang J., Jin F. (2018). Halogenated and Parent Polycyclic Aromatic Hydrocarbons in Vegetables: Levels, Dietary Intakes, and Health Risk Assessments. Sci. Total Environ..

[28] Masuda M., Wang Q., Tokumura M., Miyake Y., Amagai T. (2019). Simultaneous Determination of Polycyclic Aromatic Hydrocarbons and Their Chlorinated Derivatives in Grilled Foods. Ecotoxicol. Environ. Saf..

[29] Ni H.-G., Guo J.-Y. (2013). Parent and Halogenated Polycyclic Aromatic Hydrocarbons in Seafood from South China and Implications for Human Exposure. J. Agric. Food Chem..

[30] Ding C., Ni H.-G., Zeng H. (2012). Parent and Halogenated Polycyclic Aromatic Hydrocarbons in Rice and Implications for Human Health in China. Environ. Pollut..

[31] Ding C., Ni H.-G., Zeng H. (2013). Human Exposure to Parent and Halogenated Polycyclic Aromatic Hydrocarbons via Food Consumption in Shenzhen, China. Sci. Total Environ..

[32] Xia Z., Idowu I., Marvin C., Thomas P.J., Johnson W., Francisco O., Stetefeld J., Crimmins B., Fry M., Tomy G.T. (2019). Identification of Halogenated Polycyclic Aromatic Hydrocarbons in Biological Samples from Alberta Oil-Sands Region. Chemosphere.

[33] Huang C., Xu X., Wang D., Ma M., Rao K., Wang Z. (2018). The Aryl Hydrocarbon Receptor (AhR) Activity and DNA-Damaging Effects of Chlorinated Polycyclic Aromatic Hydrocarbons (Cl-PAHs). Chemosphere.

[34] Kido T., Sakakibara H., Ohura T., Guruge K.S., Kojima M., Hasegawa J., Iwamura T., Yamanaka N., Masuda S., Sakaguchi M. (2013). Evaluation of Chlorinated Benz[a]Anthracene on Hepatic Toxicity in Rats and Mutagenic Activity in Salmonella Typhimurium. Environ. Toxicol..

[35] Ohura T., Morita M., Makino M., Amagai T., Shimoi K. (2007). Aryl Hydrocarbon Receptor-Mediated Effects of Chlorinated Polycyclic Aromatic Hydrocarbons. Chem. Res. Toxicol..

[36] Sun J.-L., Zeng H., Ni H.-G. (2013). Halogenated Polycyclic Aromatic Hydrocarbons in the Environment. Chemosphere.

[37] Luo Y., Zhang B., Geng N., Sun S., Song X., Chen J., Zhang H. (2022). Transcriptomics and Metabolomics Analyses Provide Insights into the Difference in Toxicity of Benzo[a]Pyrene and 6-Chlorobenzo[a]Pyrene to Human Hepatic Cells. Sci. Total Environ..

[38] Luo Y., Geng N., Sun S., Cheng L., Chen S., Zhang H., Chen J. (2023). Integration Approach of Transcriptomics and Metabolomics Reveals the Toxicity of Anthracene and Its Chlorinated Derivatives on Human Hepatic Cells. Sci. Total Environ..

[39] Stein L.S., Stoica G., Tilley R., Burghardt R.C. (1991). Rat Ovarian Granulosa Cell Culture: A Model System for the Study of Cell-Cell Communication during Multistep Transformation. Cancer Res..

[40] Wickrama-Arachchige A.U.-K., Hirabayashi T., Imai Y., Guruge K.S., Dharmaratne T.S., Ohura T. (2020). Accumulation of Halogenated Polycyclic Aromatic Hydrocarbons by Different Tuna Species, Determined by High-Resolution Gas Chromatography Orbitrap Mass Spectrometry. Environ. Pollut..

[41] Zemirli N., Morel E., Molino D. (2018). Mitochondrial Dynamics in Basal and Stressful Conditions. Int. J. Mol. Sci..

[42] Cheng Q., Liu Q.Q., (Alex) Lu C. (2024). A State-of-the-Science Review of Using Mitochondrial DNA Copy Number as a Biomarker for Environmental Exposure. Environ. Pollut..

[43] Kozhukhar N., Alexeyev M.F. (2023). 35 Years of TFAM Research: Old Protein, New Puzzles. Biology.

[44] Youle R.J., Narendra D.P. (2011). Mechanisms of Mitophagy. Nat. Rev. Mol. Cell Biol..

[45] Kang R., Zeh H.J., Lotze M.T., Tang D. (2011). The Beclin 1 Network Regulates Autophagy and Apoptosis. Cell Death Differ..

[46] Wan W., Hua F., Fang P., Li C., Deng F., Chen S., Ying J., Wang X. (2022). Regulation of Mitophagy by Sirtuin Family Proteins: A Vital Role in Aging and Age-Related Diseases. Front. Aging Neurosci..

[47] Livak K.J., Schmittgen T.D. (2001). Analysis of Relative Gene Expression Data Using Real-Time Quantitative PCR and the 2^−ΔΔCT^ Method. Methods.

[48] Niu Y.-J., Zhou D., Zhou W., Nie Z.-W., Kim J.-Y., Oh Y., Lee S.-R., Cui X.-S. (2020). Nitric Oxide-Induced Protein S-Nitrosylation Causes Mitochondrial Dysfunction and Accelerates Post-Ovulatory Aging of Oocytes in Cattle. J. Anim. Reprod. Biotechnol..

[49] Heo G., Sun M.-H., Jiang W.-J., Li X.-H., Lee S.-H., Guo J., Zhou D., Cui X.-S. (2022). Rotenone Causes Mitochondrial Dysfunction and Prevents Maturation in Porcine Oocytes. PLoS ONE.

[50] Schindelin J., Arganda-Carreras I., Frise E., Kaynig V., Longair M., Pietzsch T., Preibisch S., Rueden C., Saalfeld S., Schmid B. (2012). Fiji: An Open-Source Platform for Biological-Image Analysis. Nat. Methods.

[51] Miller W.L. (2017). Disorders in the Initial Steps of Steroid Hormone Synthesis. J. Steroid Biochem. Mol. Biol..

[52] Ahmed Selim N., Wojtovich A.P. (2025). Mitochondrial Membrane Potential and Compartmentalized Signaling: Calcium, ROS, and Beyond. Redox Biol..

[53] Zhao C., Li C., Wang C., Li Y., Yang R., Zhang Q., Jiang G. (2022). Ultrasensitive Determination of 39 Parent and Emerging Halogenated Polycyclic Aromatic Hydrocarbons in Human Serum. Anal. Methods.

[54] Li W., Wu S. (2023). Challenges of Halogenated Polycyclic Aromatic Hydrocarbons in Foods: Occurrence, Risk, and Formation. Trends Food Sci. Technol..

[55] Wang Y., Li S.-H., Yang S.-J., Li X.-Q., Liu L., Ma X., Niu D., Duan X. (2023). Exposure to Phenanthrene Affects Oocyte Meiosis by Inducing Mitochondrial Dysfunction and Endoplasmic Reticulum Stress. Cell Prolif..

[56] Lee S., Hong S., Liu X., Kim C., Jung D., Yim U.H., Shim W.J., Khim J.S., Giesy J.P., Choi K. (2017). Endocrine Disrupting Potential of PAHs and Their Alkylated Analogues Associated with Oil Spills. Environ. Sci. Process. Impacts.

[57] Perono G.A., Tomy T., Loudon K., Jamshed L., Garlisi B., Lauks S., Lockington C., Ruan C., Tomy G.T., Petrik J.J. (2025). Sulfur-Containing Heterocyclic Aromatic Hydrocarbons Alter Estrogen Metabolism and Cause DNA Damage and Apoptosis in Granulosa Cells. Int. J. Mol. Sci..

[58] Xu C., Chen J.-A., Qiu Z., Zhao Q., Luo J., Yang L., Zeng H., Huang Y., Zhang L., Cao J. (2010). Ovotoxicity and PPAR-Mediated Aromatase Downregulation in Female Sprague–Dawley Rats Following Combined Oral Exposure to Benzo[a]Pyrene and Di-(2-Ethylhexyl) Phthalate. Toxicol. Lett..

[59] Zajda K., Gregoraszczuk E. (2020). Environmental Polycyclic Aromatic Hydrocarbons Mixture, in Human Blood Levels, Decreased Oestradiol Secretion by Granulosa Cells via ESR1 and GPER1 but Not ESR2 Receptor. Hum. Exp. Toxicol..

[60] Yin S., Tang M., Chen F., Li T., Liu W. (2017). Environmental Exposure to Polycyclic Aromatic Hydrocarbons (PAHs): The Correlation with and Impact on Reproductive Hormones in Umbilical Cord Serum. Environ. Pollut..

[61] Huang J., Zhang Y., Fang L., Xi F., Tang C., Ou K., Wang C. (2024). Chronic Exposure to Low Levels of Phenanthrene Induces Histological Damage and Carcinogenic Risk in the Uterus of Female Mice. Environ. Sci. Pollut. Res..

[62] Gowkielewicz M., Lipka A., Zdanowski W., Waśniewski T., Majewska M., Carlberg C. (2024). Anti-Müllerian Hormone: Biology and Role in Endocrinology and Cancers. Front. Endocrinol..

[63] Pellatt L., Hanna L., Brincat M., Galea R., Brain H., Whitehead S., Mason H. (2007). Granulosa Cell Production of Anti-Müllerian Hormone Is Increased in Polycystic Ovaries. J. Clin. Endocrinol. Metab..

[64] Yang Q., Zhao Y., Qiu X., Zhang C., Li R., Qiao J. (2015). Association of Serum Levels of Typical Organic Pollutants with Polycystic Ovary Syndrome (PCOS): A Case–Control Study. Hum. Reprod..

[65] Huang X., Chen J., Yu Y., Zhou X., Huang Y., Chen Z., Wu M., Zheng Y., Xu X., Zheng X. (2025). The Impact of Polycyclic Aromatic Hydrocarbon Metabolites on Reproductive Hormone Levels and Follicle Count in Patients with Polycystic Ovary Syndrome: A Case-Control Study. Int. J. Environ. Health Res..

[66] Chen W., Zhao H., Li Y. (2023). Mitochondrial Dynamics in Health and Disease: Mechanisms and Potential Targets. Signal Transduct. Target. Ther..

[67] Lyu Y., Yang J., Cheng L., Li Z., Zheng J. (2023). Benzo(a)Pyrene-Induced Mitochondrial Respiration and Glycolysis Disturbance in Human Neuroblastoma Cells. J. Toxicol. Sci..

[68] Hoffmann R.F., Zarrintan S., Brandenburg S.M., Kol A., de Bruin H.G., Jafari S., Dijk F., Kalicharan D., Kelders M., Gosker H.R. (2013). Prolonged Cigarette Smoke Exposure Alters Mitochondrial Structure and Function in Airway Epithelial Cells. Respir. Res..

[69] Chen W., Xu X., Wang L., Bai G., Xiang W. (2015). Low Expression of Mfn2 Is Associated with Mitochondrial Damage and Apoptosis of Ovarian Tissues in the Premature Ovarian Failure Model. PLoS ONE.

[70] Wang L., Song S., Liu X., Zhang M., Xiang W. (2019). Low MFN2 Expression Related to Ageing in Granulosa Cells Is Associated with Assisted Reproductive Technology Outcome. Reprod. Biomed. Online.

[71] Han T., Zhao Y., Jiao A., Sun Z., Zhang H., Zhao D., Wang H., Gao Q. (2025). OPA1 Deficiency Induces Mitophagy through PINK1/Parkin Pathway during Bovine Oocytes Maturation. Theriogenology.

[72] Luo Y., Zhang B., Geng N., Sun S., Song X., Chen J., Zhang H. (2022). Insights into the Hepatotoxicity of Pyrene and 1-Chloropyrene Using an Integrated Approach of Metabolomics and Transcriptomics. Sci. Total Environ..

[73] Sreerangaraja Urs D.B., Wu W.-H., Komrskova K., Postlerova P., Lin Y.-F., Tzeng C.-R., Kao S.-H. (2020). Mitochondrial Function in Modulating Human Granulosa Cell Steroidogenesis and Female Fertility. Int. J. Mol. Sci..

[74] Zhang M., Bener M.B., Jiang Z., Wang T., Esencan E., Scott R., Horvath T., Seli E. (2019). Mitofusin 2 Plays a Role in Oocyte and Follicle Development, and Is Required to Maintain Ovarian Follicular Reserve during Reproductive Aging. Aging.

[75] Zong Y., Li H., Liao P., Chen L., Pan Y., Zheng Y., Zhang C., Liu D., Zheng M., Gao J. (2024). Mitochondrial Dysfunction: Mechanisms and Advances in Therapy. Signal Transduct. Target. Ther..

[76] Pavanello S., Dioni L., Hoxha M., Fedeli U., Mielzynska-Svach D., Baccarelli A.A. (2013). Mitochondrial DNA Copy Number and Exposure to Polycyclic Aromatic Hydrocarbons. Cancer Epidemiol. Biomark. Prev..

[77] Pieters N., Koppen G., Smeets K., Napierska D., Plusquin M., Prins S.D., Weghe H.V.D., Nelen V., Cox B., Cuypers A. (2013). Decreased Mitochondrial DNA Content in Association with Exposure to Polycyclic Aromatic Hydrocarbons in House Dust during Wintertime: From a Population Enquiry to Cell Culture. PLoS ONE.

[78] Wong J.Y., Hu W., Downward G.S., Seow W.J., Bassig B.A., Ji B.-T., Wei F., Wu G., Li J., He J. (2017). Personal Exposure to Fine Particulate Matter and Benzo[a]Pyrene from Indoor Air Pollution and Leukocyte Mitochondrial DNA Copy Number in Rural China. Carcinogenesis.

[79] Kang I., Chu C.T., Kaufman B.A. (2018). The Mitochondrial Transcription Factor TFAM in Neurodegeneration: Emerging Evidence and Mechanisms. FEBS Lett..

[80] Ekstrand M.I., Falkenberg M., Rantanen A., Park C.B., Gaspari M., Hultenby K., Rustin P., Gustafsson C.M., Larsson N.-G. (2004). Mitochondrial Transcription Factor A Regulates mtDNA Copy Number in Mammals. Hum. Mol. Genet..

[81] Gannon A.M., Stämpfli M.R., Foster W.G. (2013). Cigarette Smoke Exposure Elicits Increased Autophagy and Dysregulation of Mitochondrial Dynamics in Murine Granulosa Cells. Biol. Reprod..

[82] Li A., Gao M., Liu B., Qin Y., Chen L., Liu H., Wu H., Gong G. (2022). Mitochondrial Autophagy: Molecular Mechanisms and Implications for Cardiovascular Disease. Cell Death Dis..

[83] Zhang J., Xiang H., Liu J., Chen Y., He R.-R., Liu B. (2020). Mitochondrial Sirtuin 3: New Emerging Biological Function and Therapeutic Target. Theranostics.

[84] Zhu J., Yang Q., Li H., Wang Y., Jiang Y., Wang H., Cong L., Xu J., Shen Z., Chen W. (2022). Sirt3 Deficiency Accelerates Ovarian Senescence without Affecting Spermatogenesis in Aging Mice. Free Radic. Biol. Med..

[85] Zhang M., Bener M.B., Jiang Z., Wang T., Esencan E., Scott R., Horvath T., Seli E. (2019). Mitofusin 1 Is Required for Female Fertility and to Maintain Ovarian Follicular Reserve. Cell Death Dis..

[86] Cai C., Qian J., Xiang S., Wang C., Ji Y., Cui J., Jia J. (2024). A Cytotoxicity and Mechanistic Investigation of Mono- and Di-Chloro Naphthalenes. Chemosphere.

[87] Havelock J.C., Rainey W.E., Carr B.R. (2004). Ovarian Granulosa Cell Lines. Mol. Cell. Endocrinol..

